# Dynamic culture system advances the applications of breast cancer organoids for precision medicine

**DOI:** 10.1038/s41598-025-86730-4

**Published:** 2025-03-14

**Authors:** Jun Yang, Junyuan Qu, Mei Zhang, Xiang Li, Qian Jiang, Jinxiu Kang, Pan Nie, Na Jing, Xianling Wang

**Affiliations:** 1 Department of Breast Surgery, Shanxi Province Cancer Hospital/Shanxi Hospital Affiliated to Cancer Hospital, Chinese Academy of Medical Sciences, Cancer Hospital Affiliated to Shanxi Medical University, Taiyuan, China; 2 Chongqing Kingmed Pharma Co., Ltd., Chongqing, China; 3 Zigong Fourth People’s Hospital, Zigong, China; 4 Department of Radiotherapy, Shanxi Province Cancer Hospital/Shanxi Hospital Affiliated to Cancer Hospital, Chinese Academy of Medical Sciences, Cancer Hospital Affiliated to Shanxi Medical University, Taiyuan, China; 5 Shanxi Hospital Affiliated to Cancer Hospital, Cancer Hospital, Province Cancer Hospital, Chinese Academy of Medical Sciences, Shanxi Medical University, Taiyuan, China

**Keywords:** Experimental models of disease, Breast cancer

## Abstract

Tumor organoid-based drug sensitivity prediction is a new approach for precision medicine, which has wide applications in cancer treatment and attracts increasing attention. In the field of breast cancer, conventional organoid culture methods often require more than three weeks of culture period. The culture time greatly limits the further extension of the application scenarios of breast cancer organoids. We developed a fluid system that builds on the conventional organoid “dome” culture method, which continuously and stably supplies the nutrients for the growth of breast cancer organoids. We demonstrated that this is an effective optimization method, which can shorten the culture period of breast cancer organoids without significant changes in histological characteristics and drug sensitivity features.

**Supplementary Information:**

The online version contains supplementary material available at 10.1038/s41598-025-86730-4.

## Introduction

Breast cancer is the most common and lethal malignant tumor among women, ranking first in both incidence and mortality among female tumors^1^. Breast cancer not only causes serious physical and psychological damage to the patients, but also brings huge burden to the society. In breast cancer treatment, chemotherapy has been the main means of adjuvant therapy, which can reduce tumor size before surgery (neoadjuvant chemotherapy) or eliminate residual cancer cells after surgery (adjuvant chemotherapy)^2^. To cope with the possible tumor resistance, the combination of multiple chemotherapy drugs, though common, can cause short-term to lifelong side effects for patients, which severely affect the patients’ quality of life^3^.

Based on the above needs, tumor organoid culture technology has received widespread attention since it was proposed. Tumor organoids are cultures that have certain tissue characteristics, which are constructed by isolating cancer cells from patient tumor tissues and cultivating under 3D matrix conditions with addition of specific growth factors. Tumor organoids have similar genomic, histological and drug response characteristics to the parental tumors^4,5^. Currently, a large number of studies have utilized tumor organoids to perform chemotherapy drug sensitivity tests and obtained results similar to clinical treatment^6–8^.

The commonly used organoid culture method is the dome method, which is to mix the isolated tumor cells with matrix gel and inoculate them in a well plate for culture. The specific growth factor combination and the three-dimensional structure of the matrix gel provide support for cell proliferation and morphogenesis. This method has been proven to be able to stably culture breast cancer organoids for a long time, and achieve subsequent drug stimulation experiments^9,10^.

While the dome method can fulfill the experimental requirements in the lab, it fails to fully meet the demands of clinical practice. The tissue site, sampling method and tumor cell proportion of the sample have been proven to be important factors that determine the culture success rate and efficiency. In the case that the starting tissue is small so that it will be hard to expand quickly enough to reach the necessary cell amounts for drug sensitivity analysis, which will lead to prolonged detection cycle. Breast cancer samples also face the same situation, clinicians prefer the less invasive puncture biopsy method for sampling^11,12^. Usually, less tumor cells the punctured tissue can obtained, which will directly lead to the extension of the organoid culture cycle in the existing organoid culture system, and it is difficult to provide timely diagnosis and treatment suggestions for patients. Therefore, a new organoid culture system is urgently needed to shorten the detection cycle, and maintaining a high growth rate of breast cancer organoids is a direction worth exploring.

Developmental biology has established that embryonic growth is intricately linked to vascular development, and sufficient nutrients depend on a complex vascular system^13^. Tumor organoid culture faces the same situation, due to its relatively simple cellular composition, it cannot form the necessary vascular structure, making its nutrient acquisition only dependent on passive diffusion. Under this factor, organoids are difficult to obtain sufficient nutrient supply, and their maximum size is also limited. Currently, microfluidic systems are considered to be one of the solutions to this problem^14,15^. Therefore, we will use microfluidic systems as an entry point to provide continuous and stable nutrient supply for breast cancer organoids, in order to maintain the high-level proliferation of organoids and shorten the clinical test cycle.

## Results

### A dynamic culture system facilitates the growth of organoids

**Fig. 1:**
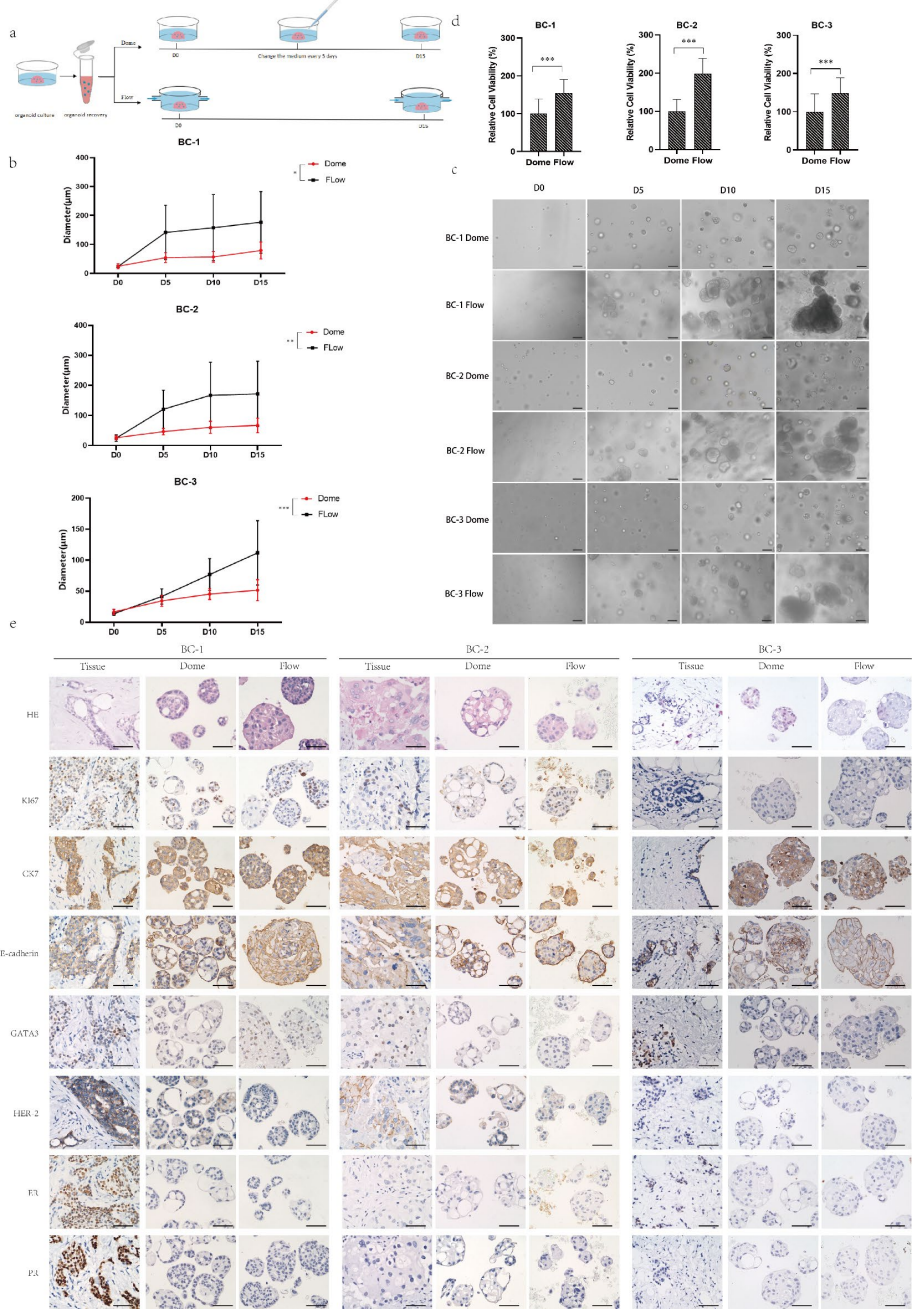
The dynamic culture system facilitates the growth of organoids. (**a**) The organoids wer*e* divided into Dome and Flow groups; the medium flow speed was 2 mL/min in the Flow group. (**b**) Diameter changes of organoids cultured for 15 days in Dome group and Flow group. (**c**) Representative microscopic pictures of organoids cultured for 15 days in Dome group and Flow group. Scale bar: 100 μm. (**d**) The relative cell viability of organoids cultured in Dome group and Flow group on day 15. (**e**) Histological and immunohistochemical images showing the organization structure and status of breast cancer-related markers (Ki67, CK7, E-cadherin, GATA3, Her-2, ER, PR) in primary tumors and organoids cultured in Dome group and Flow group for 15d. Scale bar: 50 μm. The results are expressed as the mean ± SD. Statistical significance is indicated as follows: n.s. for *P* ≥ 0.05 (not significant), * for *P* < 0.05, ** for *P* < 0.01, and *** for *P* < 0.001.

Breast cancer organoids were established from three samples of breast invasive ductal carcinoma. Then, single cell suspensions were obtained from breast cancer organoids and cultured in Matrigel using two methods: the static dome method (Dome) and the fluidic dome method (Flow) (Fig. 1a). The growth of the breast cancer organoids were continuously monitored for two weeks. By the end of the observation period, the diameters of the Flow group were significantly larger compared to the Dome group in all three samples (3/3) (Fig. 1b). Notably, the organoid morphology in the Dome group changed from solid to hollow across all the samples, whereas this phenomenon was not observed in the Flow group (Fig. 1c). Such morphological difference across two groups may complicate the analysis of proliferation rates. To address this, we measured cell viability of organoids after 15 days of culture using Alamar Blue, and found that organoids in the Flow group consistently showed higher viability than Dome group in all three samples (Fig. 1d).

Furthermore, we performed immunohistochemical staining on the organoids at the end of the observation period and compared their molecular marker expression patterns with the parental breast cancer tissue, except for estrogen receptor(ER) and progesterone receptor(PR) markers (Fig. 1e). These results demonstrated that the breast cancer organoids cultured in both Dome and Flow methods preserved the molecular characteristics of the parental tissue while enhancing organoid growth and cell viability.

### Consistent sensitivity between fluidic and static organoids

**Fig. 2:**
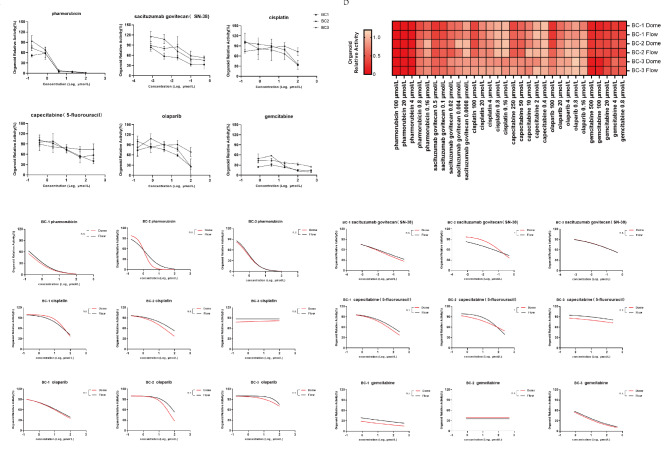
Dome group organoids and Flow group organoids exhibit similar drug sensitivity. (**a**) Drug response results for Dome Group Organoids. (**b**) Heatmap showing the mean relative survival of three groups of organoids at different drugs and concentrations. (**c**) Representative drug response curves for organoids of different drugs in the Dome group and Flow group. Differences between the two curves were compared by bilateral analysis. The results are expressed as the mean ± SD. Statistical significance is indicated as follows: ns for *P* ≥ 0.05, * for *P* < 0.05.

Drug stimulation studies were conducted on three established organoid models using olaparib, capecitabine (5’-fluorouracil substitute), cisplatin, gemcitabine, sacituzumab govitecan (SN-38 substitute), and pharmorubicin. The relative activity of drug-treated organoids showed gradual decrease when increasing drug concentrations, except for those organoids showed certain drug resistance (Fig. 2a). The three organoid models displayed a sensitivity to pharmorubicin that consistent with the clinical responses observed in patients. Of these, patient BC1 and patient BC2 were administered pharmorubicin following mastectomy, no tumor recurrence was observed during one year of follow-up. Patient BC3 received neoadjuvant therapy with pharmorubicin, and after six cycles of treatment, the tumor size reduced from 2.41 × 1.91 cm to 1.46 × 1.24 cm.

Concurrently, to assess the impact of culture protocols on organoid responsiveness to drugs, we observed the drug responses for Flow group organoids under identical conditions. This comparison revealed equivalent activity between both groups (Fig. 2b, Figure S2). Further curve fitting and the two-way ANOVA analysis comparing the groups’ responses showed 88.88% non-significant differences (Fig. 2c) suggesting similar predictability of drug reactions regardless of culture methods.

### The fluid’s mechanical effects promote breast cancer organoids growth

**Fig. 3:**
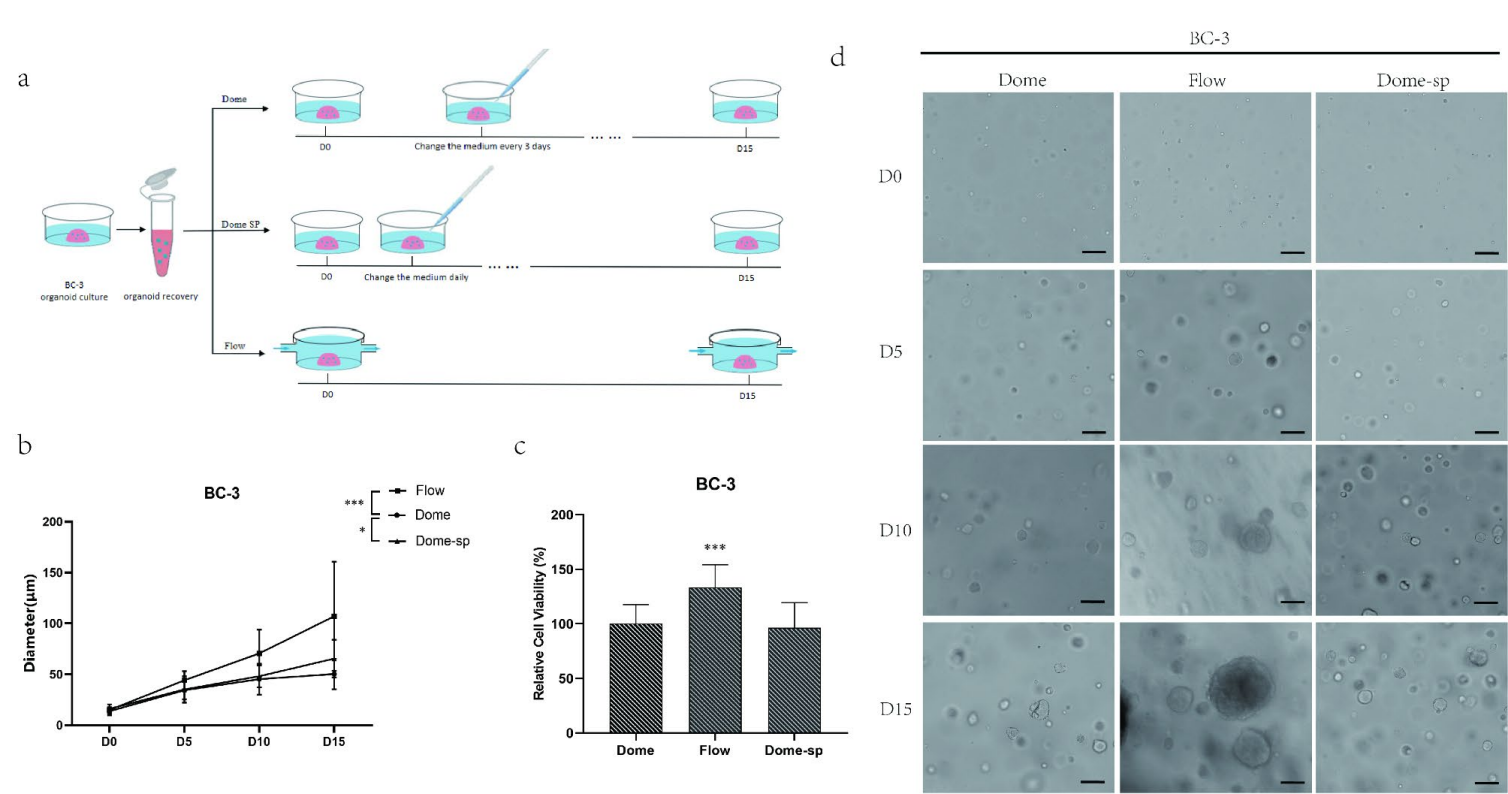
The mechanical effects of the fluid are the main factors for the increased proliferation capacity of breast cancer organoids.(**a**) The organoids wer*e* divided into Dome and Flow groups, and then according to the frequency of changed the medium, it was divided into Dome group: changed the medium every 3 days; and Dome-sp group: changed the medium every day. (**b**) Diameter changes of organoids cultured for 15 days in Dome group, Flow group and Dome-sp group. (**c**) The relative cell viability of organoids cultured in Dome group, Flow group and Dome-sp group on day 15. (**d**) Representative microscopic pictures of organoids cultured for 15 days in Dome group, Flow group and Dome-sp group. Scale bar: 100 μm. The results are expressed as the mean ± SD.Statistical significance is indicated as follows: * for *P* < 0.05 and *** for *P* < 0.001.

The fluid conditions can affect the growth of organoids in two ways: the flowing condition facilitates the maintenance of a stable nutrient supply and the influence from the fluid shear stress. To determine which factor is the primary cause of the difference in proliferation capacity, we added an extra group of dome-sp (Dome-sp) to the culture process of sample BC3, which changed the medium daily to replenish the possible material consumption (Fig. 3a).

After the 15-day observation period, the Dome-sp group did not exhibit the expected advantage in proliferation capacity, and only the Flow group showed a higher level of diameter change (Fig. 3b). Furthermore, we performed a viability assay and found that the overall cell viability of the Flow group was significantly higher than that of the Dome and Dome-sp (Fig. 3c). This indicates that the culture method used in the Dome group can fully satisfy the growth requirements of breast cancer organoids, therefore the stability of nutrients is not the factor that causes significant diameter change in our experiment.

It is noteworthy that in the Dome-sp group, we observed a general occurrence of hollow organoids (Fig. 3d). This suggests that the mechanical effects of the fluid may be the primary reason for the higher proliferation level and morphological changes in breast cancer organoids.

### Fluid shear stress alters the morphological characteristics and gene expression of breast cancer organoids

**Fig. 4:**
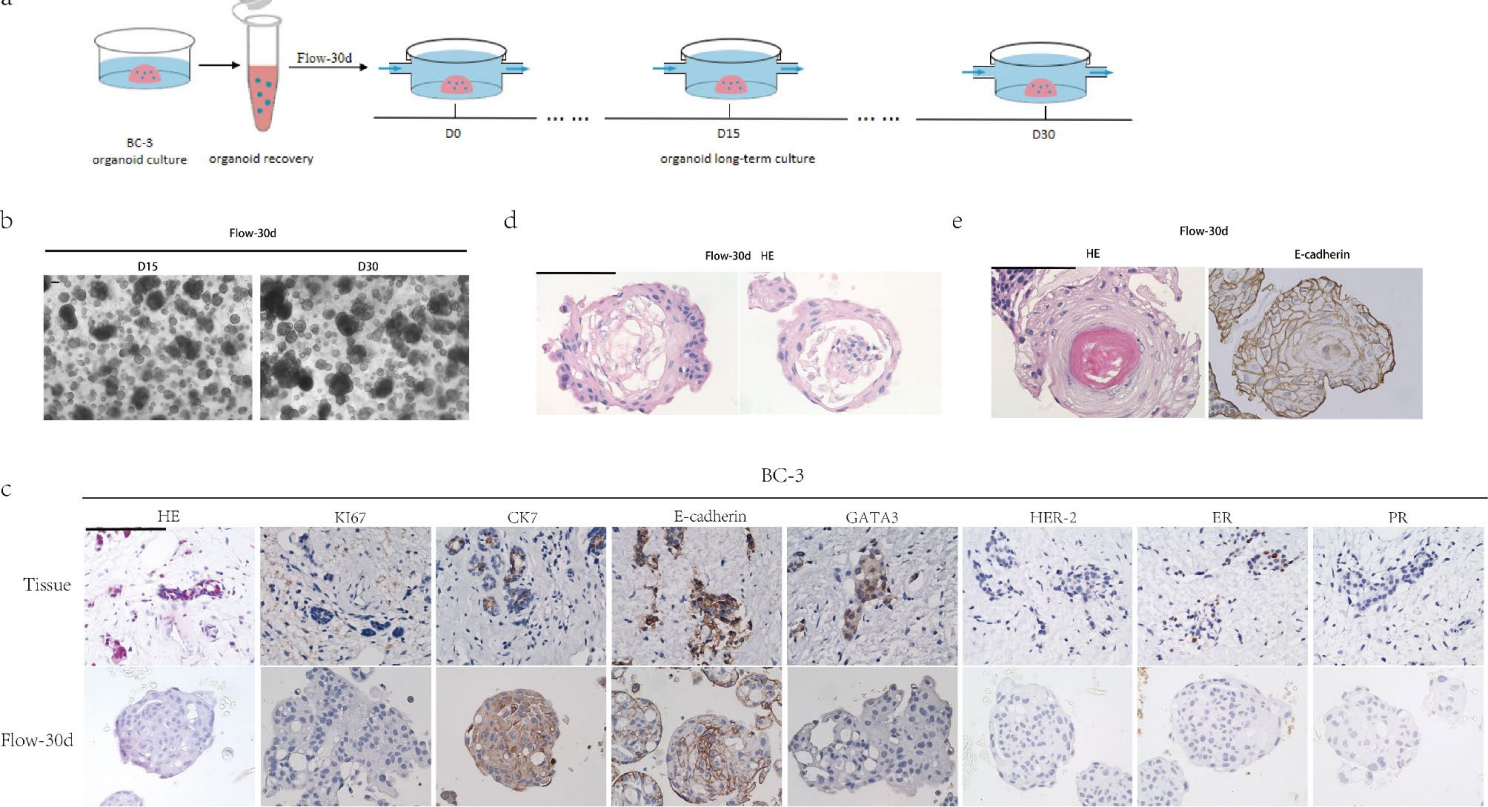
Fluid shear stress alters the morphological characteristics and gene expression of breast cancer organoids. (**a**) Extended organoids culture time in Flow group from 15 days to 30 days. (**b**) Representative microscopic images of long-term cultured organoids in Flow group. Scale bar: 200 μm. (**c**) Histological and immunohistochemical images showing the structural organization and status of breast cancer-related markers (Ki67, CK7, E-cadherin, GATA3, Her-2, ER, PR) in primary tumors and organoids cultured in Flow group for 30 days. Scale bar: 50 μm. (**d**) Representative HE image of BC-3 organoids cultured in Flow group for 30 d, detachment of cells and chromatin marginalization in the center of organoids. Scale bar: 50 μm.(**e**) Representative HE and IHC(ki67) image of BC-3 organoids cultured in Flow group for 30 d, Some organoids began to form concentric circle structures. Scale bar: 50 μm.

Furthermore, we aimed to investigate whether the hollowing observed in breast cancer organoids was a natural transformation that occurred during the prolonged culture process of breast cancer organoids, and whether the presence of fluid shear stress only delayed this process. We extended the single culture time to 30 days based on the 15-day culture cycle of the Flow group (Fig. 4a). The results showed that the central hollowing of the organoids was not observed under the microscope in the long-term culture under fluid conditions, and the organoids still showed a solid appearance (Fig. 4b). And due to the continuous increase in volume, there was a situation of mutual fusion between different organoids.

The organoids at this stage were proceeded for staining and verification. Compared with the staining results of the organoids cultured for 15 days, the organoids cultured for 30 days still exhibited molecular marker expression characteristics similar to the parental tissue, except for ER (Fig. 4c). However, we observed cell detachment and chromatin marginalization in the center of some larger organoids, with internal cells displaying typical apoptosis (Fig. 4d). Some organoids began to form concentric circle structures (Fig. 4e), and the arrangement of cells in organoid changed from the previous disorderly aggregation mode to the direction of multilayer arrangement. But none of them showed the hollow-like morphology observed in the Dome group, which indicates that the fluid shear stress may have changed the morphology of the organoids under static culture.

We tested the expression of genes related to drug resistance and proliferation, and found that the introduction of fluid conditions had a significant impact on the gene expression (Figure S3). This indicates that the fluid shear stress can induce changes in the gene expression characteristics of the organoids.

## Discussion

Tumor organoids have emerged as a cutting-edge focus in precision medicine, but the long culture time and expensive culture cost limit their applications^16^. We demonstrated that the fluid conditions can enhance the proliferation of breast cancer organoids, resulting in an increased number of cells within the same culture time. Surprisingly, the key factor in this process was not the abundant and stable supply of nutrients. There was no significant difference in diameter between the Dome group and the Dome-sp group. The results of the viability assay also confirmed this. This allows us to conclude that the existing culture system and method can be considered adequate in terms of nutrient supply.

Faithfully reproducing the basic characteristics of the parental tissue is the fundamental reason why tumor organoids are favored in the field of precision medicine. We showed that the organoids cultured under fluid conditions show consistent marker characteristics with those cultured statically, and the drug sensitivity results also show consistent responses. This indicates that the organoids cultured in fluid have the same application basis as the organoids cultured in conventional ways. Moreover, fluid conditions can effectively shorten the culture cycle and leading us to believe that the organoids cultured in fluid have a promising application prospect in the field of precision medicine.

A large number of studies have shown that under specific culture conditions, tumor organoids can be cultured and expanded stably for a long time^5,17,18^. In addition, we observed the loss of ER and PR expression, which may correlate with the aberrant activation of the NOTCH signaling pathway^19,20^. Moreover, The culture systems used in these studies typically do not exhibit fluid shear stress. In the normal human body, all types of tissue cells are continuously exposed to shear forces caused by fluid flow in the tissue microenvironments. The influence of shear force can significantly affect cell fate and plays a pivotal role in cell growth and differentiation^21,22^. The effect of shear force on gene transcription is almost inevitable, and our experimental results also show this change. We focused on several genes related to drug resistance and cellular proliferation, particularly PIM1, MCL-1, MYC, CDK6, and JAK2. PIM1 and c-Myc have been found to synergistically promote the expansion of oncological cells^21^. MCL-1 plays a regulatory role in cell differentiation and apoptosis^22^. MYC and MCL1 are co-amplified in drug-resistant breast cancer^23^. Lee et al.^24^ revealed that MYC and MCL1 cooperate to maintain cancer stem cells resistant to chemotherapy by increasing mitochondrial OXPHOS, ROS production and HIF-1α expression. Moreover, elevated levels of CDK6 and JAK2 have been observed in drug-resistant patients^23^. Our findings demonstrate that different fluidic conditions can significantly influence the expression levels of various drug-resistant genes, although their distribution is not uniform across the three samples. We noticed that organoids cultured under fluid conditions exhibited morphological characteristics different from those cultured in static culture. We observed apoptosis in the centers of larger organoids in the Flow-30 group, caused by their excessive size and insufficient internal material supply. We believe that organoids cultured in the Dome group may also encounter this problem^23^.

Hollow morphogenesis, triggered by cell movement, is a natural response of tumor cells to hostile environments, and shear stress alters this process. However, we do not consider this change to be negative. Fluid mechanical force is a common condition in tissues and is highly related to tissue and cell growth and development. We believe that the presence of fluid shear force, which induces changes, maintains the stability of the tissue structure represented by the organoids. We observed that the cell arrangement in breast cancer organoids cultured under fluid conditions for a long time has transitioned from the previous disorderly aggregation mode to a multilayer arrangement. The research results of Florian et al. reveal that this phenomenon is associated with the development and differentiation process of the mammary duct lumen^23^. Similar conclusions were also reached by Cho et al.^24^. This indicates that the presence of fluid shear force is critical and necessary for tissue reconstruction in vitro.

## Materials and methods

### Tissue collection

Breast cancer tissues were obtained from three patients with invasive ductal carcinoma(Table 1). The acquisition and use of samples for this study were reviewed and approved by the Ethics Committee of Shanxi Provincial Cancer Hospital (record number KY2023039). All research methods were conducted in accordance with approved protocols. All samples were collected with written informed consent from patients and in compliance with all relevant ethical regulations, including the Declaration of Helsinki. The patient information was de-identified before any processing and analysis of the tissue samples. The tissue samples were placed in advance DMEM/F12 (12634010, Gibco) containing 10µM Y-27,632 (HY-10071; MedChemExpress), 5% FBS (10100147, Gibco) and 100 µg/mL Primocin (ant-pm-1, InvivoGen), and transported directly to the laboratory at 2–8 °C. These cancer tissue samples were used to establish primary organoids cultures and parental tumor analysis.

**Table 1:** Clinical information for breast cancer patients.

Patient number	Age	Pathologic type	Histological grading	Molecular classification	Treatments	Response
BC-1	54	IDC	II	Luminal B	Mastectomy, postoperative chemotherapy (pharmorubicin + Cyclophosphamide + Abraxane)	No tumor recurrence detected at one year of follow-up
BC-2	74	IDC	II	HER2-positive	Mastectomy, postoperative chemotherapy (pharmorubicin + Cyclophosphamide)	No tumor recurrence detected at one year of follow-up
BC-3	49	IDC	II	Luminal B	Neoadjuvant chemotherapies (pharmorubicin + Cyclophosphamide + Abraxane)	After 6 cycles of treatment, the tumor size from 2.41*1.91 cm to 1.46*1.24 cm

1* BC, Breast Cancer; IDC, Invasive ductal carcinoma.

### Establishment and culture of breast cancer organoids

After removing the obvious connective tissue from the tissue, a piece of tissue was cut for paraffin embedding. The remaining tissue was cut into fragments of about 1 mm^3^ and dispersed using advance DMEM/F12 containing 10µM Y-27,632 and 2 mg/mL Collagenase Type I (17018029, Gibco) on a constant temperature shaker at 37 °C. The mixture was shaken for 30 min to 1 h, during which it was mixed at five-to-ten-minute intervals using a Pasteur pipette. An equal volume of advance DMEM/F12 containing 2% FBS was added to the collected mixture.After centrifugation, the cell pellet was resuspended with Matrigel (354230, Corning), and 40µL per drop was inoculated in a 24-well cell culture plate. Incubated at 37 °C for 20 min, and after the matrigel was completely solidified, added 500µL of breast cancer organoid culture medium (KCJ-7, KINGBIO) and continue to culture at 37 °C in 5% CO_2_. Change the medium every 3 days. Digest and passage organoids every 7–15 days, use 2 mg/mL Dispase II (17105041, Gibco) to recover the organoids, and use Accutase (A1110501, Gibco) to dissociate the organoids.

The breast cancer organoids used for the experiment were between the second and fifth generations. The organoids were dissociated into single cells before the experiment and were inoculated at 20,000 cells per 100 µL Matrigel. The experiments were conducted on three samples, each divided into two experimental groups, carried out simultaneously, with six replicates per group. The equipment used for fluid culture was the Quasi Vivo^®^ chamber (Kirkstall, QV500), with the equipment schematic shown in Figure S1.

### Immunohistochemistry

Tissues and organoids were fixed with 4% polyformaldehyde. Following paraffin embedding, 6-µm-thick sections were cut and fixed with Citrate Antigen Retrieval solution(C1032, Solarbio). The sections were washed 3 times in PBS (5 min each), treated with 0.3% H_2_O_2_ in distilled water, blocked and stained. The antibodies used for immunohistochemistry at their respective dilutions were as follows: PR (Abcam, ab32085) at 1:100, ER (Abcam, ab108398) at 1:200, HER2 (Abcam, ab134182) at 1:1000, CK7 (Abcam, ab181598) at 1:8000, GATA3 (Abcam, ab199428) at 1:500, E-cadherin (Abcam, ab40772) at 1:500, and Ki-67 (Abcam, ab15580) at 1:1000. Following these, secondary antibodies included Goat anti-Rabbit IgG (31460, Thermo Fisher) at 1:500. Images were acquired using Nikon ECLIPSE E100 microscope MshOt MS60, and image processing was performed using Adobe Photoshop.

### Gene expression analysis

Gene expression levels were quantified by RT-qPCR. Total RNA was extracted using Trizol reagent (beyotime, R0016), reverse transcription was performed using PrimeScript RT Master Mix (TaKaRa, RR036A), the qPCR reactions were performed using TB Green Advantage qPCR premixes (TaKaRa, 639676). MYC-for: CCTACCCTCTCAACGACAGC, MYC-rev: CTCTGACCTTTTGCCAGGAG, MCL1-for: AGAAAGCTGCATCGAACCAT, MCL1-rev: CCAGCTCCTACTCCAGCAAC, CDK6-for: CCGTGGATCTCTGGAGTGTT, CDK6-R: CTCAATTGGTTGGGCAGATT, GAPDH-for: GACAGTCAGCCGCATCTTCT, GAPDH-R: TTAAAAGCAGCCCTGGTGAC.

### Organoid proliferation assays

The organoid proliferation assay was performed using alamar blue (YEASEN, 40202ES80). The reagent was added to the organoid culture medium in the required proportion, with a reaction time of 3 h. For detection, the mixed solution was pipetted into a 96-well plate. Matrigel drops without cells were inoculated in a 24-well plate as a negative control, and a 100% reduced Alamar blue solution was used as a positive control. The absorbance values were measured at 595 nm and 630 nm, and the reduction rate was calculated according to the manufacturer’s instructions.

Organoid diameter measurements. Randomly select 3 fields of view under a 100x field of view, measure and count all clearly visible organoids within the field of view. Measure every five days.

### Drug screen

Drug sensitivity analysis of organoids was performed using CellTiter-Glo (Promega, G9241).These drugs are: olaparib(HY-10162,MCE), capecitabine (5’-fluorouracil substitute)(100187,National Institutes for Food and Drug Control), cisplatin(S1166,selleck), gemcitabine(100622,National Institutes for Food and Drug Control), sacituzumab govitecan (SN-38 substitute)(HY-13704,MCE), and pharmorubicin(130560,National Institutes for Food and Drug Control)0.2 mg/mL Dispase II was used to recover the organoids, accutase was used to dissociate the organoids.Resuspended cells in the organoid culture medium containing 5% Matrigel and inoculated them into a 384-well plate at a density of 1,000 cells per well.After incubating overnight, added the drug solution. Cell viability was assayed using CellTiter-Glo 3D (Promega) according to the manufacturer’s instructions following 3 days of drug incubation. Data analyses were performed using GraphPad Prism 8.0.2 (GraphPad Software, San Diego, California, USA, www.graphpad.com), and the dose response curves were calculated by applying nonlinear regression (curve fit).

### Statistical analyses

Data were analyzed as the mean ± SD. Normality was tested with the Shapiro-Wilk test. Comparative analysis used Two-tailed Student’s t-test, Wilcoxon’s test and Kruskal–Wallis test. *p* < 0.05 indicates significant, unless otherwise stated. The number of asterisks indicate different levels of statistical significance, (*p < 0.05; **p < 0.01; and ***p < 0.001). All data analyses and graphics were performed using GraphPad Prism 8.0.2.

## Electronic supplementary material

Below is the link to the electronic supplementary material.

Supplementary Material 1

## Data Availability

The author confirms that the key parts of the research results, such as the class organ diameter and the drug sensitivity, can be obtained in the supplementary materials of the article. Other data content can be obtained according to reasonable requirements from the corresponding authors.
