# Supplementary material for: Dynamic culture system advances the applications of breast cancer organoids for precision medicine
Source: Sci Rep. 2025 Mar 14;15:8852. doi: 10.1038/s41598-025-86730-4 (PMC11909168; doi:10.1038/s41598-025-86730-4)
Supplement: Supplementary file 1 — Supplementary Material 1 [file 41598_2025_86730_MOESM1_ESM.docx]

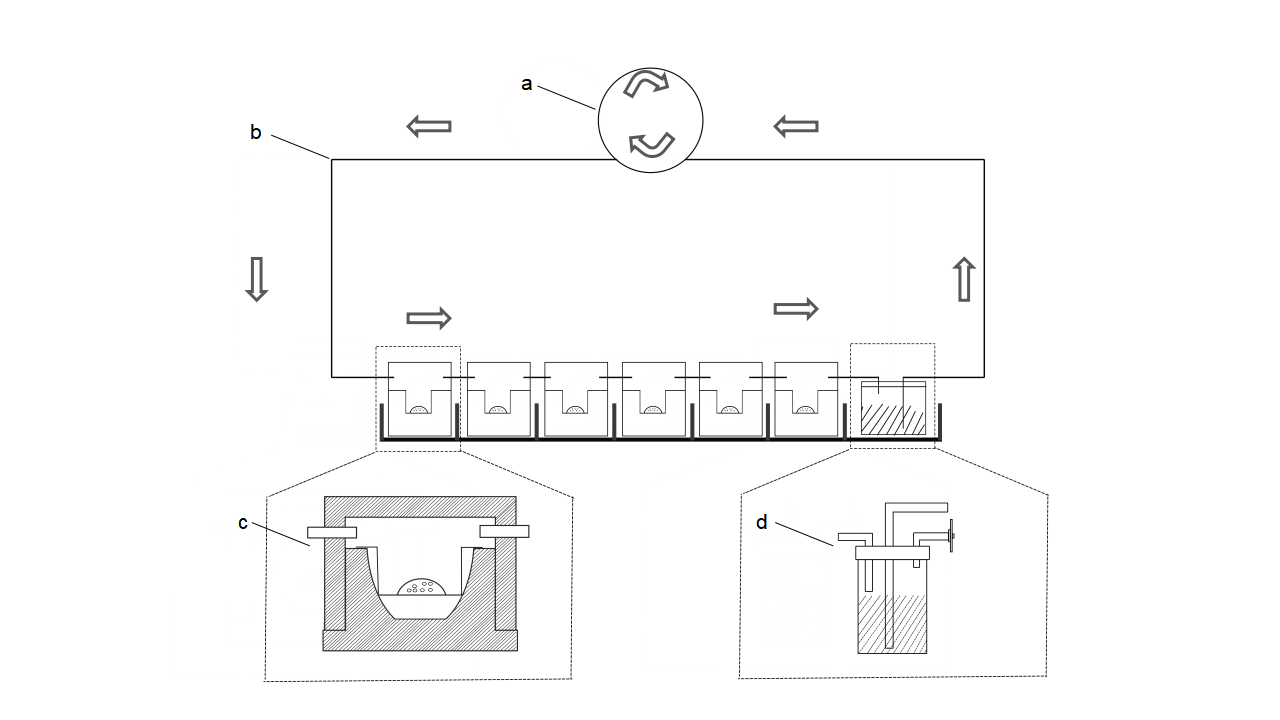


**Figure S1:** **Schematic of microfluidic system. a) Peristaltic pump；b) Pipeline; c) Organoids culture chamber; d) Medium reservoir**


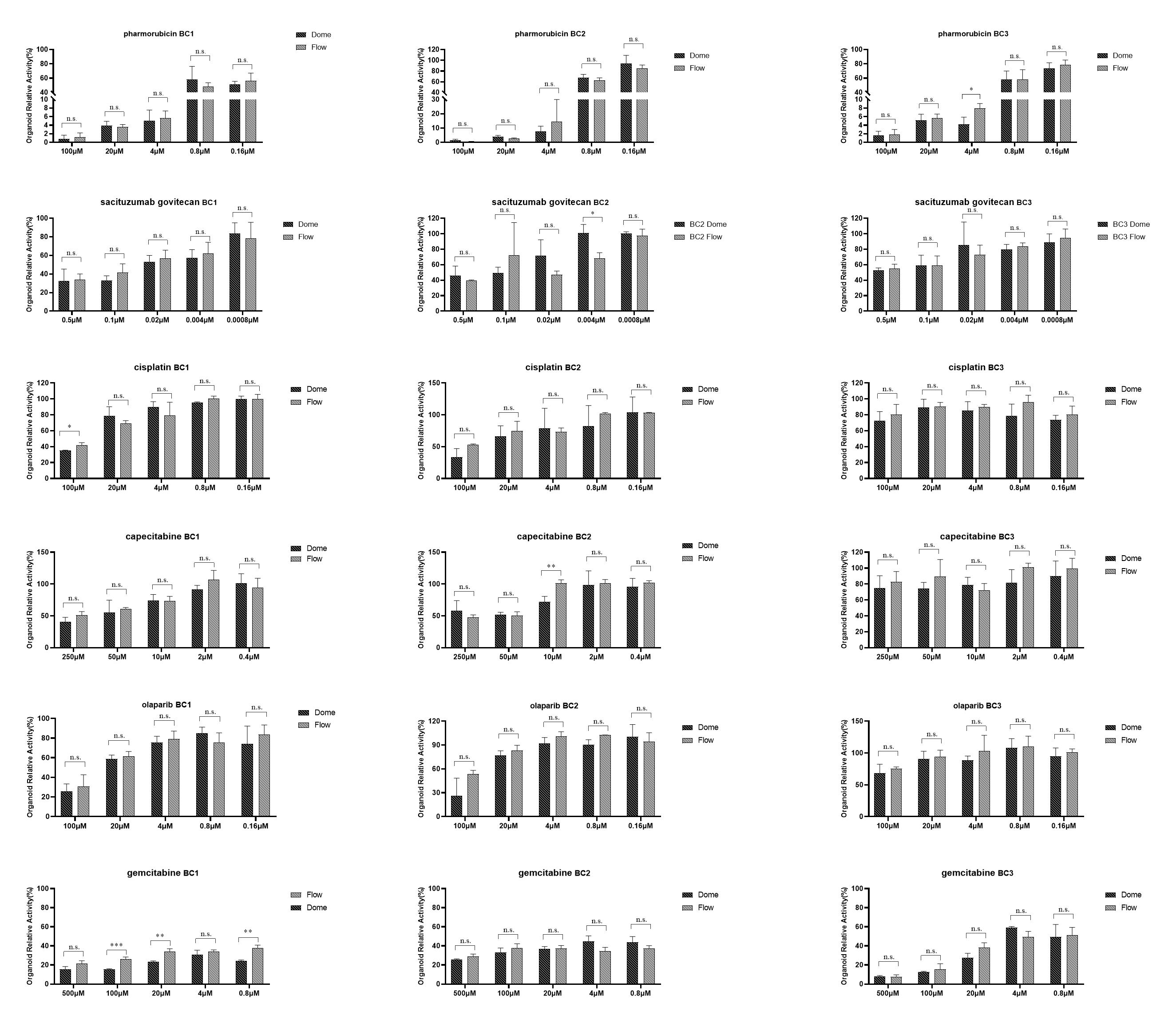


Figure S2:Drug response results for Dome group and Flow group organoids. the results are expressed as the mean±SD. Statistical significance is indicated as follows: n.s. for *P*≥0.05 (not significant), * for *P* < 0.05.


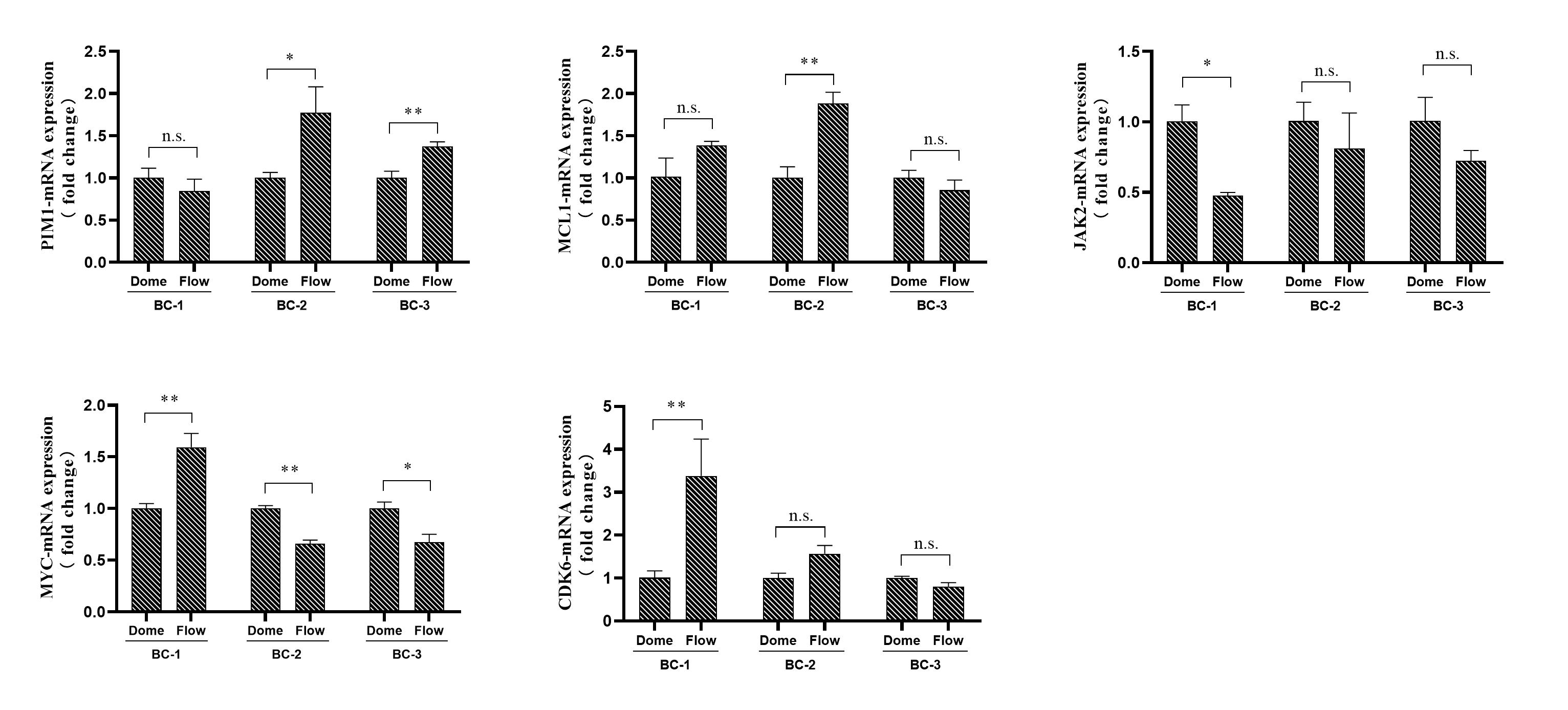


Figure S3:Relative gene expression levels of organoids cultured in Dome group and Flow group on day 15. Normalization was performed using the Dome group data.the results are expressed as the mean±SD. Statistical significance is indicated as follows: n.s. for *P*≥0.05 (not significant), * for *P* < 0.05, ** for *P* < 0.01.
